# Effect of intraocular surgery and ketamine on aqueous and serum cytokines

**Published:** 2007-07-12

**Authors:** Kyaw Lin Tu, Stephen B. Kaye, Gediminas Sidaras, William Taylor, Alan Shenkin

**Affiliations:** 1 St Paul's Eye Unit, Royal Liverpool and Broadgreen University Hospitals NHS Trust, Liverpool, United Kingdom; 2 Anaesthetic Department, Royal Liverpool and Broadgreen University Hospitals NHS Trust, Liverpool, United Kingdom; 3 Department of Clinical Biochemistry and Metabolic Medicine, Royal Liverpool and Broadgreen University Hospitals NHS Trust, Liverpool, United Kingdom

## Abstract

**Purpose:**

To determine the effect of intraocular surgery and anesthesia on aqueous and serum cytokines.

**Methods:**

Patients undergoing routine cataract surgery under general and local (peribulbar) anesthesia were randomized to those given general anesthetic with and without the use of ketamine and those having local anesthesia. Aqueous and serum levels of cytokines were collected at commencement of surgery and were determined by an immunoassay using multi-analyte biochip array technology at 18 h post-operatively.

**Results:**

At 18 h postoperative, all patients (37) showed significant and many fold increases in their aqueous levels of interleukin (IL)-1α, IL-1β, IL-4, IL-6, IL-8, vascular endothelial growth factor (VEGF), tumor necrosis factor (TNF)-α, interferon (IFN)-γ, epidermal growth factor (EGF), and monocyte chemotactic protein (MCP)-1. There was little to no increase in IL-2 and IL-10. Significant increases in some cytokines (EGF, IL-6, and IFN-γ) in the serum were also found (p=0.038). There were no significant differences in aqueous cytokine levels following the use of ketamine or between those patients who had general and local anesthesia (0.11<p<0.97).

**Conclusions:**

There is an aqueous and serum cytokine response following intraocular surgery whether local (peribulbar) or general anesthesia is used. Of the aqueous cytokines measured, three different patterns of responses emerged at 18 h post-cataract surgery; those that were highly increased (IL-8, IL-6, IFN-γ, and TNF-α), medium increase (IL-1β, VEGF, IL-4, and MCP-1), and those with little to no change (EGF, IL-1α, IL-2, and IL-10).

## Introduction

Intraocular surgery commonly results in postoperative intraocular inflammation. This is thought to result from trauma to ocular structures, disruption of the blood barrier, an immune hypersensitivity reaction, or an infective-immune response. This inflammatory response plays an important role in the outcome of a variety of surgical procedures, for example, it may be associated with a reduction in survival of a transplanted cornea [1], predisposition to cystoid macular edema [2], or a failure of glaucoma filtration surgery [3]. Control of the inflammatory response is, therefore, likely to be important in determining the success of surgery. Characterization of aqueous cytokines may be helpful in staging disease activity and in correlating cytokine profiles with clinical signs [4-8]. The cytokine response may be important in endothelial loss following intraocular surgery. For example, whereas IL-8 and IL-10 have roles in suppressing inflammation and aid vascular endothelial cell survival [9,10], TNF, IL-6, and IFN-γ are linked with vascular endothelial cell apoptosis [11,12]. Although there have been few studies on the cytokine response to intraocular surgery, IL-6 is likely to be a key mediator of postoperative uveitis and has been shown in both animal [13] and human [14] studies to significantly increase following intraocular surgery.

Cardiopulmonary bypass (CPB) has been proposed as a model for systemic inflammatory response syndrome [15,16]. The anesthetic agent, ketamine, has been found to attenuate the IL-6 response after cardiopulmonary bypass (CPB) [17]. Thus far, it is not known if the systemic anti-inflammatory effect of ketamine extends to the eye to cause suppression of post-ocular surgery inflammation. Although systemic (plasma) concentrations of IL-1β, TNF-α, and IL-8 have been found to increase following both general and local anesthesia for cataract surgery [18], these, as well as other aqueous cytokines, have not been measured in the aqueous humor following intraocular surgery. Therefore, we sought to characterize the cytokine response to intraocular surgery using cataract surgery as the model and to determine whether ketamine had a similar effect found with systemic inflammation.

## Methods

### Patients

Patients undergoing routine uncomplicated cataract surgery either under general or local (peribulbar) anesthesia were enrolled into the study after obtaining their informed consent. The decision whether surgery was to be carried out under general or local anesthesia had been determined independently by the patient's ophthalmologist and was unrelated to potential inclusion in this study. The study protocol was approved by the local Research Ethics Committee and the Declaration of Helsinki was adhered to. Patients were excluded if they were using or had used immunosuppressive medication, were using topical ophthalmic medication, had a history of autoimmune disease or other systemic inflammatory disease within the past 5 years, or had a known hypersensitivity to ketamine. Patients were not excluded if they had controlled, non-insulin dependent diabetes mellitus and had not had or required retinal laser treatment. Patients were randomly selected from three groups: those undergoing cataract surgery under general anesthesia with ketamine (GA+ or group 1), those without ketamine (GA- or group 2), and those having local (peribulbar) anesthesia without ketamine (LA or group 3).

### Surgical procedure

Standard small incision (3.2 mm) cataract surgery using phacoemulsification was performed by four experienced cataract surgeons. Preoperative assessment of cataract density was made by a single surgeon using the LOCSIII grading system [19]. The length of the procedure, phacoemulsification time and power, and untoward events during the procedure such as iris touch or hemorrhage were recorded. All patients received the same preoperative topical medication, namely guttae cyclopentolate 1% and phenylephrine 2.5%. A subconjunctival injection of gentamicin 5 mg was given at the end of the procedure. Towards the end of the study, patients received intracameral vancomycin (1 mg in 0.1 ml) in line with changed departmental antimicrobial prophylaxis protocol for cataract surgery. Postoperative topical medication was a reducing regime of guttae betamethasone 0.4% and chloramphenicol 0.5%. Postoperative treatment was not commenced until the 18 h sample had been collected.

### Anesthetic procedure

All patients had cataract surgery on a Thursday afternoon theatre list (2-5 p.m.). General anesthesia was induced intravenously with a bolus dose of propofol 1-2 mg/Kg and was maintained using volatile vapor of isoflurane 1% in an oxygen and nitrous oxide mixture. Fentanyl (1 μg/Kg) provided analgesia and atracurium (0.5 mg/Kg) was used to provide neuromuscular blockade and to facilitate ventilation and oxygenation. Ketamine (1 mg/Kg) was administered by an I.V. infusion over the duration of the surgery. Postoperatively, cocodamol was administered orally every six h, as required, to alleviate pain. Local anesthesia was administered by a peribulbar injection of 5 ml of a mixture of Lignocaine 2% and Levobupivacaine 0.5%.

### Sample Collection

Aqueous and serum samples were collected on the first postoperative morning (9-11 a.m.), approximately 18 h from the time of their cataract surgery. Five milliliters of venous blood and 100 μl of aqueous (obtained through the cataract side port incision) were obtained at the commencement of surgery and prior to the administration of ketamine. The side port incision was placed anterior to the limbus to avoid any bleeding. The postoperative samples (aqueous and venous blood) were collected at 18 h postoperatively. The postoperative aqueous sample was collected as follows. Following topical anesthesia with guttae proxymethocaine, the side port site was dabbed with a cotton bud dipped in a 5% betadine solution. Using slit lamp biomicroscopy, a Pierce hydrodissection cannula attached to a 1 ml tuberculin syringe was inserted by one operator into the side port incision flush with the internal opening into the anterior chamber. Then, the second operator withdrew 30-50 μl of aqueous humor; the amount of aqueous withdrawn was determined by monitoring the depth of the anterior chamber. All samples were stored at -80 °C for subsequent analysis.

### Clinical outcome measures

Intraocular inflammation was assessed preoperatively and at 18 h by grading the anterior chamber flare (F) from one to four and counting the number of cells (C) in a 0.2 mm beam under 25X magnification. Complications during sampling, postoperative complications, and visual acuity of patients were recorded.

### Analysis of samples

Levels of interleukins (IL) -1α, IL-1β, IL-2, IL-4, IL-6, IL-8, IL-10, vascular endothelial growth factor (VEGF), tumor necrosis factor-α (TNF-α), interferon-γ(IFN-γ), epidermal growth factor (EGF), and monocyte chemotactic protein-1 (MCP-1) were measured using a Biochip Array System [20] (Evidence Investigator, Randox Ltd, Co. Antrim, N. Ireland). The biochip is a solid substrate with multiple specific cytokine antibodies attached at predefined sites on the surface. Each cytokine binds to its specific antibody then appropriate enzyme-labeled second antibodies are added and the cytokines are quantified by chemiluminescence using a CCD camera and imaging system. Samples were thawed immediately before analysis, diluted 1:3 if necessary to ensure a volume greater than 100 μl, and 100 μl of the samples were applied directly to each reaction well in the Biochip. Samples were analyzed in two batches where all samples from the same patient were analyzed in the same batch. The between-batch variability for all analytes was less than 12% and within-batch variability less than 10%. As there is little information available on the aqueous concentration of some of the cytokines, it was not possible to determine the most appropriate dilution for sample analysis.

### Statistical Analysis

For some of the cytokines, it was not possible to determine the lower or upper values due to lack of sufficient sample for further dilution or concentration. Therefore, the concentrations were ranked, a Friedman test for related samples was used to compare the three groups, and a Kolmogorov-Smirnov test to compare pre to postoperative levels using SPSS (version 14.1) statistical program. A correction factor for multiple testing was made to alpha where indicated.

## Results

Fifty-two patients were enrolled into the study. Fifteen patients withdrew from the study prior to taking of second sample and were excluded. Eleven patients (mean age 69 years, female:male=7:4), 11 patients (mean age 74 years, female:male=9:2) and 15 patients (mean age 75 years, female:male=9:6) were categorized into groups 1, 2, and 3, respectively, giving 37 sets of samples (Table 1).

**Table 1:** Pre and postoperative aqueous cytokine response following cataract surgery.

**Patient**	**Grp**	**Cataract grade**	**EPT**	**Inflammation**	**IL-2**		**IL-4**		**IL-6**		**IL-8**		**IL-10**		**VEGF**	
					**Pre**	**Post**	**Pre**	**Post**	**Pre**	**Post**	**Pre**	**Post**	**Pre**	**Post**	**Pre**	**Post**
1	GA-	NO3NC3	0.528	F1C2	10.9	28.0	10.5	80.1	1.00	>350	10	1480	1.0	1.0	276	2356
2*	GA-	NO2NC2	1.656	F1C1	44.2	10.3	36.5	28.3	350	>350	28	1633	30.6	1.0	98	1633
3	GA-	NO1NC1	0.168	F1C0-1	15.7	31.9	6.8	32.2	1	>350	10	383	1.4	1.0	9	286
4	GA-	NO2NC3C2	1.008	F2C2	0	0	5.8	9.8	1.00	>350	17	951	1.8	1.0	27	103
5	GA-	NO3NC3C4	0.672	F1C2	0	0	5.2	33.2	3	>350	30	772	0	1.0	27	400
6*	GA-	NO3NC3P2	0.696	F2C2	0	0	4.4	18.8	121	>350	165	1722	35.7	1.0	25	211
7	GA-	NO1NC1	0.336	F1C1	0	0	8.4	19.4	>350	>350	127	810	1.0	1.0	140	370
8	GA-	NO3NC3C2	1.224	F2C2	0	0	5.4	34.3	224	>350	62	413	1.0	1.0	76	300
9	GA-	NO2NC3	0.936	F1C1	0	0	4.0	9.8	6	>.350	10	740	1.0	1.0	35	452
10	GA-	NO3NC3	0.528	F1C2	0	0	9.8	24.5	3	>350	48	325	1.0	1.0	38	21
11	GA-	NO2NC2C2	0.504	F1C1	0	0	8.1	20.9	7	>350	10	1655	1.0	1.0	36	230
12*	GA+	NO3NC3	0.48	F1C0/1*	46.6	27.5	8.3	54.8	96	>350	10	1488	1.0	7.3	136	180
13	GA+	NO2NC2P2	0.288	F2C1	20.5	40.6	5.9	33.4	1.00	>350	10	845	5.9	1.1	15	254
14	GA+	C2P4	0.456	F2C2	9.0	30.8	7.2	30.1	1.00	>350	10	1871	1.0	1.0	125	584
15	GA+	NO3NC3	0.696	F1C1	43.7	13.8	4.5	21.9	5	>350	10	541	1.0	1.0	146	259
16	GA+	NO2NC2	1.08	F1C1	1.1	38.8	8.0	24.7	4	>350	10	186	1.0	1.0	39	350
17	GA+	NO1NC1P3	0.312	F1C2	38.4	1	13.7	64.6	20	>350	10	1600	1.6	1.0	91	1542
18	GA+	NO3NC3C4	1.488	F2C2	0	0	5.0	52.4	3	>350	10	614	1.0	1.0	29	70
19	GA+	NO1NC1P2	0.24	F1C1	0	0	6.8	29.4	16	>350	17	602	1.0	1.0	27	247
20	GA+	NO2NC2	0.24	F1C1	0	0	4.4	23.0	1.00	>350	10	777	2.5	1.0	39	160
21	GA+	NO4NC4	0.48	F2C1	11.0	1.00	24.8	40.3	5	210	10	525	4.6	5.8	0	0
22	GA+	NO3NC3	1.08	F1C2	31.8	33.40	1.9	21.6	350	3010	35	140	0.5	5.8	352	540
23	LA	NO2NC2	0.456	F1C1	46.8	50.6	3.7	14.8	<1	350	10	715	1.00	6.1	18	290
24	LA	NO3NC3P4	0.528	F1C1	75.2	8.0	4.5	73.8	5	>350	10	3285	1.00	1.0	44	252
25	LA	NO2NC2C2	0.392	F1C1	47.5	54.0	4.2	34.0	<1	>350	10	853	1.00	1.0	14	358
26	LA	NO4NC4	1.44	F1C3	60.1	46.1	3.0	42.7	11	>350	10	1295	1.00	1.0	66	349
27	LA	NO1NC1C2	0.384	F1C2	39.2	45.3	6.3	27.6	<1	>350	10	1029	2.61	1.0	35	235
28	LA	NO1NC1P2	0.288	F1C1	7.5	15.3	10.7	33.4	4	>350	10	430	1.00	1.0	43	527
29	LA	NO2NC3	0.456	F1C2	2.0	1.00	7.2	51.9	8	>350	10	1371	4.7	1.0	69	802
30	LA	NO2NC2	0.84	F1C1	0	0	9.8	16.3	196	>350	22	556	3.7	1.0	47	1542
31	LA	NO2NC3	0.384	F2C1	0	0	3.1	15.9	<1	>350	48	294	1.0	1.0	33	204
32	LA	NO2NC2	1.464	F1C1	0	0	0	36.0	0	>350	0	552	0	1.1	0	558
33	LA	NO2NC2P2	0.432	F1C1	13.1	10.7	23.2	33.9	126	210	14	468	4.0	5.3	0	0
34	LA	NO2NC2	0.84	F1C2	43.4	8.40	23.40	6.50	221	>350	26	1379	0	1.1	0	438
35	LA	NO1NC2	0.24	F1C1	2.90	0	5.90	6.40	11	>350	6	1132	0	1.6	11	263
36	LA	No2NC2	0.504	F1C2	14.0	14.4	12.60	9.7	26	>350	11	772	0.60	0.9	0	287
37	LA	NO3NC3	0.408	F1C1	18.5	14.4	10.30	6.50	170	>350	19	2203	0.20	0.9	33	239
**Patient**	**Grp**	**Cataract grade**	**EPT**	**Inflammation**	**IFN**		**TNFalpha**		**IL-1a**		**IL-1b**		**MCP-1**		**EGF**	
					**Pre**	**Post**	**Pre**	**Post**	**Pre**	**Post**	**Pre**	**Post**	**Pre**	**Post**	**Pre**	**Post**
1	GA-	NO3NC3	0.528	F1C2	0.3	3.8	1.0	23.9	6.7	21.7	7.7	25.5	191	>1200	<1.0	<1.0
2*	GA-	NO2NC2	1.656	F1C1	9.1	25.9	18.9	14.8	11.4	3.5	<1.0	18.3	1979	>1200	<1.0	2.8
3	GA-	NO1NC1	0.168	F1C0-1	5.6	16.8	<1.0	28.7	1.9	10.0	<1.0	13.6	419	>1200	<1.0	0
4	GA-	NO2NC3C2	1.008	F2C2	0.3	34.3	<1.0	11.5	<1.0	2.0	1.5	11.6	206	>1200	<1.0	11.3
5	GA-	NO3NC3C4	0.672	F1C2	0.2	51.1	3.46	7.4	<1.0	<1.0	<1.0	15.3	325	>1200	<1.0	0
6*	GA-	NO3NC3P2	0.696	F2C2	0.1	52.7	<1.0	24.7	26	3.6	19.1	6.3	479	>1200	<1.0	28.6
7	GA-	NO1NC1	0.336	F1C1	2.4	34.4	<1.0	42.7	<1.0	1.8	<1.0	16.3	1269	>1200	<1.0	10.2
8	GA-	NO3NC3C2	1.224	F2C2	0	20.9	4.09	10.9	<1.0	0.7	1.2	16.4	486	>1200	<1.0	0
9	GA-	NO2NC3C2	0.936	F1C1	1.0	35.7	2.84	27.3	<1.0	2.4	<1.0	10.5	337	>1200	<1.0	0
10	GA-	NO3NC3	0.528	F1C2	2.2	4.1	<1.0	<1.0	<1.0	6.9	1.2	2.2	298	>1200	<1.0	0
11	GA-	NO2NC2C2	0.504	F1C1	5.6	16.5	<1.0	39.4	<1.0	6.6	<1.0	7.4	196	>1200	<1.0	0
12*	GA+	NO3NC3	0.48	F1C0/1*	0.10	22.2	5.39	45.5	8.0	18.1	2.2	32.3	520	>1200	<1.0	1.6
13	GA+	NO2NC2P2	0.288	F2C1	6.8	21.4	2.07	24.7	10.9	5.9	<1.0	13.0	230	>1200	<1.0	2.3
14	GA+	C2P4	0.456	F2C2	5.0	34.1	1.66	17.9	3.2	9.1	<1.0	12.0	258	>1200	<1.0	<1.0
15	GA+	NO3NC3	0.696	F1C1	2.4	6.1	4.78	13.4	3.3	8.5	<1.0	11.1	434	>1200	<1.0	<1.0
16	GA+	NO2NC2	1.08	F1C1	1.7	2.9	<1.0	4.2	10.4	10.0	3.7	2.7	190	1146	<1.0	<1.0
17	GA+	NO1NC1P3	0.312	F1C2	2.2	34.3	4.48	29.8	7.5	7.5	1.1	41.1	279	>1200	<1.0	<1.0
18	GA+	NO3NC3C4	1.488	F2C2	2.1	28.5	<1.0	16.5	<1.0	<1.0	1.2	15.4	314	>1200	<1.0	<1.0
19	GA+	NO1NC1P2	0.24	F1C1	1.3	16.5	<1.0	25.5	<1.0	2.9	1.1	5.3	219	>1200	<1.0	<1.0
20	GA+	NO2ND2	0.24	F1C1	1.1	15.5	<1.0	<1.0	<1.0	<1.0	<1.0	8.5	124	>1200	<1.0	<1.0
21	GA+	NO4NC4	0.48	F2C1	0	0	7.3	57.6	5.23	5.23	<1.0	<1.0	209	>679	11.3	16.8
22	GA+	NO3NC3	1.08	F1C2	7.00	23.2	0.0	0.0	7.00	21.30	<1.0	5.70	927	1384	0.0	0.0
23	LA	NO2ND2	0.456	F1C1	0.10	5.4	<1.0	14.6	11.9	12.5	<1.0	3.6	151	>1200	<1.0	2.51
24	LA	NO3NC3P4	0.528	F1C1	0.78	0.9	3.0	12.6	6.4	502.8	1.3	129.3	393	>1200	<1.0	<1.0
25	LA	NO2NC2C2	0.392	F1C1	0.10	10.9	4.1	47.4	2.8	15.2	<1.0	26.5	119	>1200	<1.0	<1.0
26	LA	NO4NC4	1.44	F1C3	1.1	24.8	<1.0	46.2	8.0	8.6	1.5	26.5	451	>1200	<1.0	<1.0
27	LA	NO1NC1C2	0.384	F1C2	0.7	22.6	2.4	31.7	10.4	3.8	1.9	24.8	111	>1200	<1.0	<1.0
28	LA	NO1NC1P2	0.288	F1C1	0.10	<0.1	<1.0	15.8	10	<1.0	1.2	8.8	331	2906	<1.0	<1.0
29	LA	NO2NC3C2	0.456	F1C2	4.0	9.6	3.6	48.1	2.8	2.8	<1	24.2	279	>1200	<1.0	<1.0
30	LA	NO2 NC2	0.84	F1C1	25.9	29.8	<1.0	7.1	<1.0	<1.0	3.6	5.1	395	>1200	<1.0	2.15
31	LA	NO2NC3	0.384	F2C1	0.1	12.2	<1.0	<1.0	2.2	<1.0	<1.0	10.1	416	>1200	<1.0	<1.0
32	LA	NO2NC2	1.464	F1C1	0	9.70	0	30.6	0	<1.0	0	9.0	0	>1200	<1.0	<1.0
33	LA	NO2NC2P2	0.432	F1C1	0	0	6.8	74.6	<5.23	12.4	<1.0	<1.0	494	>679	13.7	17.0
34	LA	NO2NC2	0.84	F1C2	20.80	44.6	0.00	0	1.50	20.20	14.40	1.30	1019	1200	0	0
35	LA	NO1NC2	0.24	F1C1	7.00	90.8	1.20	0.9	2.30	78.10	1.50	3.70	269	1200	0	0
36	LA	No2NC2	0.504	F1C2	7.90	49.8	0.00	5.1	2.20	1.10	349	1200	0	0	0	0
37	LA	NO3NC3	0.408	F1C1	8.40	41.3	1.80	0	2.30	78.90	<1.0	1.10	443	1200	0	0

Patient groups: general anesthesia with (GA+) and without (GA-) ketamine, and local anesthesia (LA). Cataract classification: nuclear opalescence (NO) and nuclear color (NC), and cortical (C) and posterior subcapsular (P) lens opacities. Anterior chamber inflammation: aqueous flare (F), scale 1-4; cells (C) in the anterior chamber, that is, number of cells in a 0.2 mm beam graded as 0 (no cells), 1 (1-3 cells) and 2 (3-5 cells). Effective phacoemulsification time (EPT) is the product of average power and time. Aqueous cytokines: interleukin (IL)-1α, IL-1β, IL-4, IL-6, IL-8, vascular endothelial growth factor (VEGF), tumor necrosis factor-α (TNFα), interferon (IFN)-γ, epidermal growth factor (EGF), and monocyte chemotactic protein-1 (MCP-1). Note the comparative changes in the pre and postoperative levels of the measured aqueous cytokines such as IL-6 and IL-2. Also compare with Table 3 for serum responses of these cytokines. There were no differences between the three patient groups and no correlation with grade of cataract or EPT.

### Clinical outcome

The effective phacoemulsification time (EPT), derived from multiplying average phacoemulsification power and the phacoemulsification time, and the grade of cataract for each patient is included in Table 2 along with the degree of postoperative inflammation. No patient developed any unexpected preoperative complication. Postoperatively, 29 and 8 patients had grade 1 and 2 flare, respectively. Twenty-two, fourteen, and one patients had anterior chamber cell counts of grade 1, 2, and 3, respectively (Table 1). There was no significant difference between the EPT for the three groups, which were 0.75, 0.60, and 0.60 for groups 1, 2, and 3, respectively (p=0.57).

**Table 2:** Mean pre and postoperative aqueous cytokine leves.

**Group**	**IL-2 Pre/Post**	**IL-4 Pre/Post**	**IL-6 Pre/Post**	**IL-8 Pre/Post**	**IL-10 Pre/Post**	**VEGF Pre/Post**	**INF Pre/Post**	**TNFα Pre/Post**	**IL-1a Pre/Post**	**IL-1b Pre/Post**	**MCP Pre/Post**	**EGF Pre/Post**
GA-	24	10	97	47	7	71	2	3	5	3	562	1
	23	28	350	989	1	569	27	21	5	13	11200	5
GA+	25	8	71	13	2	98	3	3	5	1	361	2
	23	34	560	971	2	381	20	21	8	13	1167	5
LA	29	9	54	15	1	36	6	2	5	26	346	2
	21	26	341	1106	2	445	25	21	47	92	1199	2
All	27	9	72	24	3	67	4	3	5	11	416	1
	22	29	413	1029	2	463	24	21	22	44	1189	4

Patient groups: general anesthesia with (GA+) and without (GA-) ketamine, and local anesthesia (LA). Aqueous cytokines: interleukin (IL)-1α, IL-1β, IL-4, IL-6, IL-8, vascular endothelial growth factor (VEGF), tumor necrosis factor-α (TNFα), interferon (IFN)-γ, epidermal growth factor (EGF), and monocyte chemotactic protein-1 (MCP-1). It should be noted that to determine the approximate mean values, a cut-off value at either the lower or higher concentration range was used. The values in this table should, therefore, only be considered as an approximated concentration. Although there were large differences in the mean pre to postoperative aqueous cytokine responses there were no significant differences between the three patient groups.

### Complications of sampling

One patient each from groups 1 and 2 complained of discomfort and headache following collection of the postoperative aqueous sample at 18 h. In group 3 (LA), one patient's anterior chamber shallowed to approximately 1.5 to 2 mm during the postoperative sampling procedure but reformed satisfactorily after padding of the eye for ten min. All three patients had good visual acuities postoperatively (6/6, 6/5, and 6/9, respectively) with an uneventful clinical course.

### Aqueous cytokines

The pre and postoperative aqueous cytokine concentrations are included in Table 1.

### Preoperative levels

Apart from IL-1β and EGF, there were detectable levels of the remaining cytokines in the aqueous humor. In particular, all patients had relatively high aqueous levels of MCP-1 and VEGF compared to the remaining other cytokines analyzed. In ten patients (patients 2, 6-8, 12, 22, 30, 33, 34, and 37) aqueous but not serum levels of IL-6 were relatively raised preoperatively (Table 1). Of these ten patients, two had atrophic macular degeneration (one other patient with dry age-related macular degeneration did not have raised aqueous IL-6), two had non-insulin dependent diabetes mellitus, one had had removal of a pterygium three years prior to cataract surgery and one had had a trabeculectomy six years prior to cataract surgery.

### Postoperative aqueous cytokine concentrations

**Figure 1:**
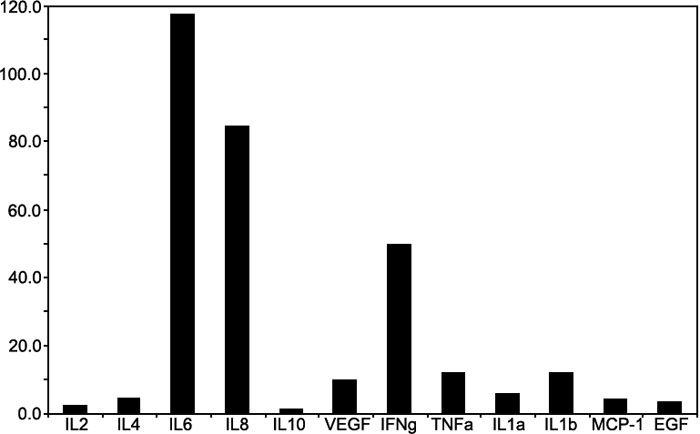
Ratio of post (18 h) to preoperative aqueous cytokine levels following cataract surgery. The post to preoperative aqueous cytokine ratio was determined using the approximated mean pre- and post-operative values. Where the end point of the measured cytokine could not be determined, the value at the limit of detection was used. Aqueous cytokines: interleukin (IL)-1α, IL-1β, IL-4, IL-6, IL-8, vascular endothelial growth factor (VEGF), tumor necrosis factor-α (TNFα), interferon (IFN)-γ, epidermal growth factor (EGF), and monocyte chemotactic protein-1 (MCP-1).

Some of the cytokine measurements showed little to no increase while others showed many fold increases. There were significant differences between all of the pre and postoperative aqueous cytokine levels (0.003<p<0.001) except perhaps for IL-2 (p=0.07) and IL10 (p=0.05) especially if alpha is adjusted to 0.005. There were, however, no differences in the pre to postoperative cytokine levels between the three patient groups, GA+, GA-, and LA, for all of the 12 cytokines measured (0.069<p<0.886). Additionally, there was no difference in the change in aqueous cytokines between patients who had general and local anesthesia (0.11<p<0.97). The pre to postoperative changes in concentration could be approximately grouped into three semi-quantitative classes: those showing little to no increase, EGF, IL-1α, IL-2, and IL-10; medium increase, IL-1β, VEGF, IL-4, and MCP-1; and those showing a high (many fold) increase, IL-8, IL-6, IFN-γ, and TNF-α. This is reflected in Figure 1 as the ratio of the post to preoperative aqueous cytokine levels (used for descriptive purposes, the lower and upper limit of detection as appropriate and included in Table 2 as the mean pre and postoperative aqueous cytokine levels). There was no apparent correlation between the EPT and the ratio of post to preoperative aqueous cytokine ratios (0.06<p<0.87).

### Serum cytokine levels

**Figure 2:**
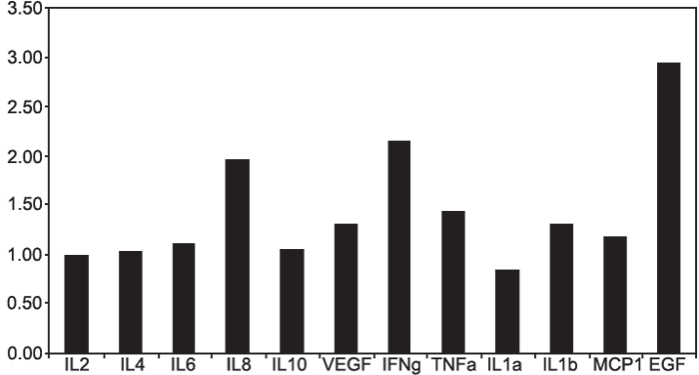
The post to preoperative serum cytokine ratio was determined using the approximated mean pre- and post-operative values. Where the end point of the measured cytokine could not be determined, the value at the limit of detection was used. Serum cytokines: interleukin (IL)-1α, IL-1β, IL-4, IL-6, IL-8, vascular endothelial growth factor (VEGF), tumor necrosis factor-α (TNFα), interferon (IFN)-γ, epidermal growth factor (EGF), and monocyte chemotactic protein-1 (MCP-1).

The preoperative and postoperative serum levels for the cytokines measured are shown in Figure 2 (ratio of the post to preoperative serum cytokine levels) and included in Table 3. There were small but significant increases in IFN-γ (p=0.005), IL-6 (p=0.02), IL-8 (p=0.03), VEGF (p=0.003), and TNF-α (p=0.013). Although, if alpha is adjusted to 0.005 to correct for multiple testing (ten cytokines), these effects can only be considered preliminary. For the aqueous concentrations, there were no significant differences in the pre and postoperative serum levels between the three groups (0.20<p<0.96). Three patients (two GA-, one GA+) had relatively raised preoperative serum but not aqueous IL-6 levels. No systemic reasons for the raised preoperative serum IL-6 levels were identified.

**Table 3:** Pre and postoperative serum cytokine response following cataract surgery.

**Patient**	**IL2**		**IL4**		**IL6**		**IL8**		**IL10**		**VEGF**		**IFN**		**TNFα**		**IL1a**		**IL1b**		**MCP1**		**EGF**	
**GA-**	**Pre**	**Post**	**Pre**	**Post**	**Pre**	**Post**	**Pre**	**Post**	**Pre**	**Post**	**Pre**	**Post**	**Pre**	**Post**	**Pre**	**Post**	**Pre**	**Post**	**Pre**	**Post**	**Pre**	**Post**	**Pre**	**Post**
1	0	0	0	0	14	17	21.6	30.4	0	0	577	684	0	1.6	6.5	5.9	0	0	0	0	271	265	405	500
2	0	0	0	0	8.8	12.3	8.8	18.6	0.7	1.9	221	297	0	0	5.9	6.1	0	0	0	0	247	501	232	349
3	0	1	3.1	4.5	88.7	93	12.1	13.4	0.2	0.1	174	224	0	2.8	5.2	6.7	2.7	2.1	0.4	1.8	276	321	324	248
5	0	0	0	0.8	82.9	84.1	9.1	11.1	0	0	60	84	0	0.6	2.3	4	0	0	0	0	550	558	262	334
6	6	0	0	0	0	63.9	13.1	28.6	0	0	215	272	0.6	0.8	5.2	8.7	1.9	0	0	0	281	459	156	295
7	22.2	20.5	0	0	0	0.2	0.7	0.2	0.5	0.2	20	21	2.3	2.8	4.2	6.5	1.1	0	4.4	5.7	211	162	27	72
**GA+**																								
12	244.3	249.6	0	0	3	4.5	14.4	18.3	0	0	565	656	0	1.3	9.5	11.9	0	0	109.3	109.2	527	545	54	71
14	1.9	4.4	0	0	8.6	8.9	6.3	8.3	0	0	83	63	0.8	3.5	2.8	7.96	0	0	0	0	429	505	278	298
16	3.9	3.3	3.1	3.1	160.4	173.6	10.3	13	0	0	205	252	0.6	3.3	8.2	15.3	0	0	0	0	435	362	161	298
4	0	7.5	0	0	0	0.6	9	10.2	0	0	234	457	0	0	6.5	9.8	0	0	0	0	244	340	35	35
**LA**																								
24	1.9	0	0	0	0	0	6.7	11	0	0	811	860	0	0.6	7.6	6.1	0	0	0	0	304	310	140	87
26	5	7.9	0	0	0	0.2	0.2	1.9	0	0	25	35	0	0	3.3	4.4	0	0	0	0	343	352	296	214
30	1.9	1.9	0	0	0	0.5	7.9	7.9	0	0	34	57	1.1	5.6	4.6	4.4	0	0	0	0	408	462	10	179

Patient groups: general anesthesia with (GA+) and without (GA-) ketamine, and local anesthesia (LA). Serum cytokines: interleukin (IL)-1α, IL-1β, IL-4, IL-6, IL-8, vascular endothelial growth factor (VEGF), tumor necrosis factor-α (TNFα), interferon (IFN)-γ, epidermal growth factor (EGF), and monocyte chemotactic protein-1 (MCP-1). Although the changes are smaller compared to the aqueous levels (Table 1), note the comparative changes in the pre and postoperative levels of the measured serum cytokines such as IL-6 and IL-2. There were no differences between the three patient groups.

## Discussion

Methodologies for studying the cellular infiltration and the presence of various cytokines in aqueous humor range from conventional assay methods like ELISA (and sequential ELISA) to techniques such as flow cytometric multiplex array (CBA) [21,22] and multiple bead immunoassay [8]. More recently, biochips using protein array technology have made possible, simultaneously, detection of multiple analytes, reduction in sample and reagent volumes, and high output of test results. Intra- and inter-assay imprecision for most of the analytes in this study was <10% and comparison studies have shown compatible results with other methods for measurement of cytokines [20]. Because the samples were batch tested and there was a limited amount of sample (40 μl), it was not possible to determine the optimum dilution for each cytokine. This resulted in the difficulty in determining the precise concentration for some cytokines, hence, there was a cut-off, for example, at either less than 0.1 or greater than 350 pg/ml. Nevertheless the post to preoperative changes were often many fold greater. Although every attempt was made to immediately store the samples, it is possible that there was degradation of the cytokines prior to storage and as the sample warmed, its concentration was altered. The ratio of post to preoperative concentrations should, however, be reflective of the surgical effect. Although it would have been useful to measure total protein concentration, all of the aqueous samples were used up to meet the volume requirements for the Biochip assays. While it is likely that there may have been a change in aqueous protein concentration from preoperative to postoperative, the changes in cytokines were so great that the pre to postoperative results remain valid. Similarly, the cytokine concentrations relative to each would be the same.

The 18 h postoperative time point was chosen because it was the most convenient for the patient and also because cytokine concentrations based on IL-6 have been found to be high at this time [17]. Clearly, because only two time points were sampled, little inferences can be made. Nevertheless, of the 12 cytokines analyzed in this study, all except EGF were detectable in the aqueous preoperatively and all except IL-2 and IL-10 showed significant increases 18 h following surgery. In all of the study groups, aqueous cytokine levels broadly showed three different patterns of response at 18 h postoperatively: those showing a high increase, IL-8, IL-6, IFN-γ, and TNF-α; those showing a medium increase, IL-1β, VEGF, IL-4, and MCP-1; and those showing little or no increase, EGF, IL-1α, IL-2, and IL-10. Thus, as far as aqueous levels are concerned, predominantly macrophage-derived cytokines (TNF-α, IL-1β, IL-6, IL-8, and MCP1) and predominantly endothelial cell-derived cytokines (VEGF, TNF-α, IL-6, IL-8, and MCP) showed several fold increases. This may be reflecting a breakdown in the blood-aqueous barrier. The results for IL-6 are similar to those of Malacaze et al who reported low to undetectable preoperative aqueous IL-6 levels with several fold increases three days following extracapsular cataract surgery with no detectable IL-6 bioactivity in the serum [14].

General anesthesia using Fentanyl, Thiopentone, Isoflurane, Propofol, and nitrous oxide has been associated with an increase in IFN-γ, IFN-α, TNF-α, and soluble interleukin-2 receptor synthesis, but not in IL-1β and IL-6 [23]. Anesthetic technique can modify the balance of cytokines associated with surgery [24,25]. Ketamine, an intravenous anesthetic agent, had been found to have suppressive effects on macrophage functions including inflammatory cytokine production possibly via reduction of mitochondrial membrane potential in cell culture [26]. It has also been shown to suppress staphylococcal enterotoxin B-induced proinflammatory cytokine production in human whole blood [27]. In our study, patients having surgery under GA received a bolus injection of Propofol or an infusion of 1 mg/Kg of ketamine. We found no suppressive effect of Propofol on TNF-α levels as has been reported in studies on rats [28]. Similarly, with regard to ketamine, the absence of a detectable effect on aqueous or serum cytokine responses may reflect poor penetration of ketamine into the eye, peculiarity of eye surgery, or insufficient therapeutic levels. Although in rats the minimum effective dose to inhibit IL-6 secretion is between 5-10 mg/Kg [29,30], the dose used in our study (1 mg/Kg) was higher than that used by Roytblat et al. (0.25 mg/Kg) who noted an attenuation of postoperative serum IL-6 levels in the patients given ketamine in a study on patients undergoing cardio-pulmonary bypass [17]. Although cataract surgery would not be expected to cause a major systemic insult, serum cytokine levels also showed a slight but significant increase following surgery, reflecting some systemic effect or release into the circulation. We could not detect any differences between general or local anesthesia in terms of the clinical or cytokine response. Although increases in IL-1β, IL-6, and IL-8 are attenuated by high doses of lidocaine in high concentrations (0.5 mg/ml) [31], this level is unlikely to be reached in the anterior chamber following peribulbar injection.

It has previously been reported that greater phacoemulsification energy is required for higher grades of nuclear color (NC) and nuclear opalescence (NO) as graded by the Lens Opacities Classification System, version III (LOCSIII) [32]. Although not a primary outcome measure and therefore speculative, the absence of a detectable correlation between aqueous cytokine response and effective phacoemulsification time/energy suggests that the aqueous cytokine response at 18 h bears little correlation with the amount of phacoemulsification energy within the range used. Although the inflammatory cytokine response may be relatively standard for this type of surgery, it may be more marked in complicated or difficult surgical cases thus indicates further work in such cases.

In the absence of additional time point measurements, it is difficult to speculate on the significance of the cytokine responses measured. That is, although our results represent snapshots of aqueous cytokine levels pre and 18 h post cataract surgery, the cytokine response following corneal infection [33] or trauma [34] is intense and usually short-lived. Whether the aqueous levels of these cytokines remain elevated postoperatively is clearly important for further surgery such as penetrating keratoplasty following cataract or glaucoma filtration surgery. It would be useful to measure these cytokines as well as TGF-β at other postoperative time points, particularly later time points, to determine the response pattern and maintenance of the response. Although there is between subject variation, the response (at least at 18 h) appears reasonably uniform so that different patient groups could be selected for different time intervals which would mean repeated individual testing is unnecessary. In addition, it would be informative to measure the cytokine response in cases where the postoperative inflammatory response has been greater than expected and to correlate or standardize with the total aqueous protein concentration. In conclusion, we have found that aqueous cytokine levels showed three different patterns of response at 18 h post-cataract surgery; those that were highly increased (IL-8, IL-6, IFN-γ, and TNF-α), intermediately increased (IL-1β, VEGF, IL-4, and MCP-1), and those with little to no change (EGF, IL-1α, IL-2, and IL-10). This response appears to be unaffected by the use of ketamine.
